# Improvement of physical, chemical, and biological properties of aridisol from Botswana by the incorporation of torrefied biomass

**DOI:** 10.1038/srep28011

**Published:** 2016-06-17

**Authors:** Tatsuki Ogura, Yasuhiro Date, Masego Masukujane, Tidimalo Coetzee, Kinya Akashi, Jun Kikuchi

**Affiliations:** 1 RIKEN Center for Sustainable Resource Science, 1-7-22 Suehiro-cho, Tsurumi-ku, Yokohama 230-0045, Japan; 2 Graduate School of Medical Life Science, Yokohama City University, 1-7-29 Suehiro-cho, Tsurumi-ku, Yokohama 230-0045, Japan; 3 Department of Agricultural Research, Ministry of Agriculture, Private Bag 0033, Gaborone, Botswana; 4 Faculty of Agriculture, Tottori University, 4-101 Koyama-cho, Tottori 680-8533, Japan; 5 Graduate School of Bioagricultural Sciences, Nagoya University, 1 Furo-cho, Chikusa-ku, Nagoya 464-0810, Japan

## Abstract

Effective use of agricultural residual biomass may be beneficial for both local and global ecosystems. Recently, biochar has received attention as a soil enhancer, and its effects on plant growth and soil microbiota have been investigated. However, there is little information on how the physical, chemical, and biological properties of soil amended with biochar are affected. In this study, we evaluated the effects of the incorporation of torrefied plant biomass on physical and structural properties, elemental profiles, initial plant growth, and metabolic and microbial dynamics in aridisol from Botswana. Hemicellulose in the biomass was degraded while cellulose and lignin were not, owing to the relatively low-temperature treatment in the torrefaction preparation. Water retentivity and mineral availability for plants were improved in soils with torrefied biomass. Furthermore, fertilization with 3% and 5% of torrefied biomass enhanced initial plant growth and elemental uptake. Although the metabolic and microbial dynamics of the control soil were dominantly associated with a C1 metabolism, those of the 3% and 5% torrefied biomass soils were dominantly associated with an organic acid metabolism. Torrefied biomass was shown to be an effective soil amendment by enhancing water retentivity, structural stability, and plant growth and controlling soil metabolites and microbiota.

In African dryland landscapes, improving nutrient-poor soils is important for increasing agricultural productivity, particularly because a significant population growth is expected in this region over the next 100 years. In the Republic of Botswana in southern Africa, *Jatropha curcas* L. has received attention as a biomass resource^1^,^2^ although has exhibited unsatisfactory growth due to the arid climate, chilling injury, and oligotrophic soil conditions (aridisols)^3^,^4^. Therefore, methods of soil amendment are expected to promote its agricultural production in nonfarming lands.

In dryland ecosystems, such as arid African landscapes, termites, which build termite mounds, play a key role in soil amelioration^5^. Their effects may be artificially achieved through soil amendment using charcoal-like soil enhancers^6^,^7^. Charcoal has a porous structure and harbors soil microbes^8^ that play roles in soil enrichment. Activated charcoal has been reported to increase nutrients, reduce nutrient leaching, enhance nutrient uptake, and increase crop production^9^,^10^. Recently, biochar, which is made from post-harvest biomass residues, has been studied for its use to amend soils in various African countries^11^,^12^.

Torrefied biomass, which is a kind of biochar made at low temperature under anaerobic conditions, is made by the torrefaction of plant biomass derived from grasses and/or woods. Torrefied biomass revealed isothermal pyrolyzed biomass at relatively low temperature ranges of 200 °C–300 °C^13^,^14^. The treatment evaporates the internal water from the biomass with an economic use of energy^15^; therefore, this type of biochar exploits this resource of carbon-rich material. Watanabe *et al.* characterized torrefied *Jatropha* biomass components, and they suggest that detoxification of phorbol ester by thermal degradation renders it suitable as a soil amendment^16^.

The beneficial effects of biochar on plant growth and soil microbiota have also been investigated^12^,^13^,^17^,^18^. For example, Anders *et al.*^19^ reported that biochar enhanced the positive correlation between nutrients and microbiota more in nutrient-poor soils than in nutrient-rich soils. Fox *et al.*^20^ also reported that soil amended with biochar enhanced the microbially mediated nutrient mobilization of S and P resulting in an improvement in plant growth. However, the relationship between the metabolites and microbiota has not been evaluated in soil amended with biochar.

A soil study focused on the humic substance or microbial properties in forest soils using nuclear magnetic resonance (NMR)^21^,^22^. However, a comprehensive approach based on physical, chemical, and biological viewpoints was greatly anticipated to evaluate the effects of biochar on soil amendment. We had evaluated various soil properties, such as plant degrading abilities, using some analytical strategies and compared NMR and other meta-analytical methods^23^,^24^. In this study, we focused on torrefied biomass and evaluated the soil amendment effects of this biomass in an aridisol from Botswana. We evaluated water retentivity, chemical components, effect on plant growth, and metabolic and microbial variations in the soil (Fig. 1).

## Results and Discussion

### Torrefication profile of *J. curcas*

To prepare the torrefied biomass, the thermal degradation profile of *J. curcas* was characterized by thermogravimetric (TG)-differential thermal analysis (DTA), attenuated total reflectance (ATR)-Fourier transform infrared (FTIR), grain size distribution, and ^1^H-^13^C heteronuclear single quantum coherence (HSQC) NMR spectra (Figs S1–S4). Dehydration occurred at approximately 70 °C and degradation of the hemicellulose components at approximately 160 °C–280 °C (Fig. S1). The torrefied biomass bonds, revealed by the vibration of the benzene C–H bond, the syringyl C–O vibration, and cellulose and hemicellulose C–H deformations that were known to cleave under a fast pyrolysis treatment^25^, were broken between 240 °C and 250 °C (Fig. S2; Table S1). The grain size of torrefied biomass was over 1,400, 710, 355, and 106 μm or smaller, and the ratios were 11.40%, 25.30%, 28.45%, 23.06%, and 11.79%, respectively (Fig. S3). The chemical component comparison of raw and 240 °C torrefied biomass showed that some signals such as adipate, d-fructose, and l-glutamine disappeared, but many saccharide signals such as maltodextrin remained (Fig. S4; Table S2). Because denaturation of the supramolecular structures with the remaining main components such as cellulose at 240 °C may be able to provide adsorption of water and nutrient elements during incorporation into soil for plant growth, the torrefication temperature in these experiments was set at 240 °C. In addition, major toxic compounds (the profiles were already characterized in a previous study^16^) were under the detection limit in the NMR spectra of biomass torrefied at 240 °C, suggesting very low concentrations of these toxins in the biomass. Therefore, we presumed that the soil community should be less affected by toxins when biomass torrefied at 240 °C was used for soil amendment experiments. Moreover, most bacteria that existed in the biomass were eliminated because the torrefaction process has the effect of dry sterilization. Thus, we considered that further analyses using soil ecosystems would be unaffected by external bacteria that existed in the biomass.

### Influence of torrefied biomass on soil

The physical properties of soils with or without torrefied biomass were evaluated by measuring the water-holding capability, soil compression stress, and relaxation time (Figs 2 and S5). The water contents of the control and 1%, 3%, and 5% torrefied biomass soils were significantly different at 30.0%, 33.3%, 34.1%, and 35.7%/volume, respectively (Fig. 2A). A similar trend in water contents was observed in soils with raw biomass (Fig. S6). Furthermore, the maximum mechanical stress values of the soils were 0.56, 1.39, 0.99, and 1.49 N, respectively (Fig. 2B). The 1% and 5% torrefied biomass soils were significantly different from the control at depths of 17.8 and 5.6 mm, respectively. The water *T*_2_ relaxation times of these soils for bound water with biomass in the soils were 76.99, 25.05, 20.16, and 11.27 ms, respectively, and significant differences between control and soils with torrefied biomass were observed (Fig. 2C). The *T*_2_ relaxation times of free water were 222.78, 62.02, 46.44, and 19.13 ms, respectively (Fig. 2D). The abundance ratios of binding vs. free water in the control and 1%, 3%, and 5% torrefied biomass soils were 63:37, 81:19, 66:34, and 79:21, respectively. The higher compression stress values and shorter *T*_*2*_ relaxation times in the torrefied biomass soils compared with the control soil suggested that torrefied biomass soils facilitated soil structural stability, resulting in higher water contents and retention capabilities. These results indicated that the physical character of soil was greatly changed by the addition of torrefied biomass and that soil water retention was improved. Although a similar effect of water retention capability was observed in soils mixed with raw biomass, the chemical properties were different between raw and torrefied biomass (Figs S1, S2 and S4). The torrefied biomass retained the cellulose component by degradation of the supramolecular structure known as lignocellulose. Therefore, one of the advantages of utilizing torrefied biomass compared with raw biomass is the easy access and exchange to energy by microbiota.

Soil elements with or without torrefied biomass were characterized by water extraction followed by HNO_3_ extraction using inductively coupled plasma-optical emission spectrometry (ICP-OES; Fig. 3). Principal component analysis (PCA) profiles showed similar trends between water and HNO_3_ extractions, meaning that the elemental profiles were different between control soils and those with added torrefied biomass (Fig. 3A,B). Some elements such as K, P, and S increased in the torrefied biomass soils compared with the control because these elements were derived from the torrefied biomass (Fig. 3C). In addition, the dissolution rates of some elements such as K, Na, and P in water compared with those in HNO_3_ were highly increased in the torrefied biomass soils compared with the control (Fig. 3D). Since water-soluble elements are readily accessible to plants, the elemental availability (especially of K, Na, and P) to plants was improved by the addition of torrefied biomass to the soils. However, many elements were largely dissolved in HNO_3_, suggesting that in nature these elements are trapped by supramolecular structures in the soil.

### Soil maturation

The metabolic dynamics of microbiota during soil maturation were evaluated using ^1^H-NMR spectra (Fig. S7). Metabolic profiles from day 0 to 21 in the soils with torrefied biomass were varied, but each profile at day 28 was similar to that of the control. This result indicated that organic components such as polysaccharides were digested during the period from day 0 to 21, and that the available organic components were finally lost by day 28 (as in the control). Moreover, the metabolic profiles of soil with fishmeal were largely varied; degradation of creatine and trimethylamine *N*-oxide (TMAO) was accompanied by the production of acetate, methylamine, dimethylamine, and trimethylamine from day 0 to 13 (Fig. S8). Creatine and TMAO are the most abundant components in fish water-soluble fractions^26^,^27^. This result indicated that the soil microbiota metabolized and utilized these fish components and produced some metabolites such as methylamine, dimethylamine, and trimethylamine, which are derivatives of TMAO. However, a lot of nutrients remained in the fishmeal soil compared with the torrefied biomass, indicating that an excess of nutrients in the fishmeal soil prevented seedling and plant growth. Therefore, only the soils with torrefied biomass were used for further analyses.

### Effect of torrefied biomass on the initial growth stage of *J. curcas*

To evaluate the effect of torrefied biomass on *Jatropha* growth, germinated *J. curcas* were transplanted to matured soils and grown for 4 weeks. The plant heights of the control and of the 1%, 3%, and 5% torrefied biomass treatments were 10.50 and 9.23, 8.75, and 8.95 cm, respectively; the stem diameters were 3.04, 3.76, 4.02, and 4.58 mm, respectively (Fig. 4A,B); and the root lengths were significantly greater by 65.81, 73.75, 93.85, and 91.31 mm, respectively (Fig. 4C). The weights of the roots, stems, and leaves also tended to increase with increasing torrefied biomass treatments (Fig. 4D). The uptake of soil elements by plants in the initial growth stages was characterized by ICP-OES (Fig. 4E). K and Na were present in higher concentrations, but Si, Mn, and Ba were lower in plants grown in torrefied biomass-amended soils than in the control. Although Na is known to inhibit plant growth, the low concentration in the soils should not affect plant growth and less than 0.04% was incorporated into the plants. In addition, it is known that excessive Mn and Ba inhibit plant growth^28^,^29^. Moreover, the carboxyl functional group in organic compounds and polysaccharides such as hemicellulose in plants and alginic acid in algae is also known to be heavy metal adsorbents^30^,^31^,^32^. The result shows that torrefied biomass has the capacity to provide beneficial minerals such as K and to inhibit toxic element absorption. Thus, torrefied biomass can be utilized as a soil conditioner for soil amendment.

### Metabolic and microbial dynamics in soil during initial plant growth

Metabolic soil dynamics during initial plant growth were evaluated using ^1^H-NMR spectra (Fig. 5A,B) in combination with 2D-J spectra for annotation (Fig. S9; Table S3). The metabolic profiles were clustered based on the differences between the control and torrefied biomass soils. In the 3% and 5% torrefied biomass soils, the profiles were PC1 positive with the factors being l-valine, lactate, acetate, and succinate. Organic acids produced by anaerobic microbes due to cellulosic degradation are known to be phosphate-solubilizing and plant growth-promoting substances^33^,^34^. Thus, the torrefied biomass was considered to effect soil fertilization and enhance plant growth. Formate decreased from week 0 to 1, and methanol and butyrate showed a similar trend increasing in weeks 1 and 3 and decreasing in week 4 in the control (Fig. S10A–C). In contrast, acetate, lactate, and succinate showed similar decreases from week 0 to 4 and l-valine increased from week 0 to 2 with 5% torrefied biomass (Fig. S10D–G).

Microbial profiles during plant growth were analyzed with a MiSeq sequencer (Fig. 5C,D). The control contributed to PC1, and 3% and 5% torrefied biomass soils contributed to PC2. In the control, the most predominant microbe was *Methylotenera* sp. In contrast, some microbes including *Opitutus* sp. and *Devosia* sp. were associated with the 3% and 5% torrefied biomass soil. *Methylobacterium* sp. and *Methylotenera* sp., which exist on plant leaves and are associated with methanol consumption and production^35^,^36^, were highly abundant, and *Methylotenera* sp. greatly increased from week 0 to 1 in the control (Fig. S11A,B). Based on methanol variations, these microbes were inferred to be dominantly associated with a C1 metabolism in the control. *Bacillus* sp., *Devosia* sp., and *Opitutus* sp. were highly abundant, and *Devosia* sp. and *Opitutus* sp. showed similar trends during plant growth in 3% and 5% torrefied biomass soils (Fig. S11C–E). *Devosia* sp. and *Opitutus* sp. are known to use lactate as a carbon source^37^,^38^, and the time-course variations were associated with acetate, lactate, and succinate dynamics. Thus, these microbes were inferred to be associated with the metabolism of these organic acids and considered to be key players in torrefied biomass adjusted soil environments for promoting plant growth. In the future, the effects of soil amendments using torrefied biomass should be evaluated by field experiments.

In conclusion, the effects of soil amended with torrefied biomass were evaluated with respect to their physical properties, initial plant growth, and metabolic and microbial dynamic soil profiles (Fig. 6). Torrefied biomass improved the physical and structural properties of soil such as water retentivity and structural stability. Soil amended with 3% and 5% torrefied biomass enhanced the initial growth of *J. curcas* in the form of increased stem diameter, root length, and element uptake ability. Although the metabolic and microbial dynamics of the control were associated with a C1 metabolism, those of the 3% and 5% torrefied biomass samples were associated with an organic acid metabolism. These results indicate that torrefied biomass is effective as a soil amendment by increasing water retentivity and structural stability, enhancing plant growth, and controlling soil metabolites and microbiota.

## Methods

### Sample preparation and experimental design

**Figure 1:**
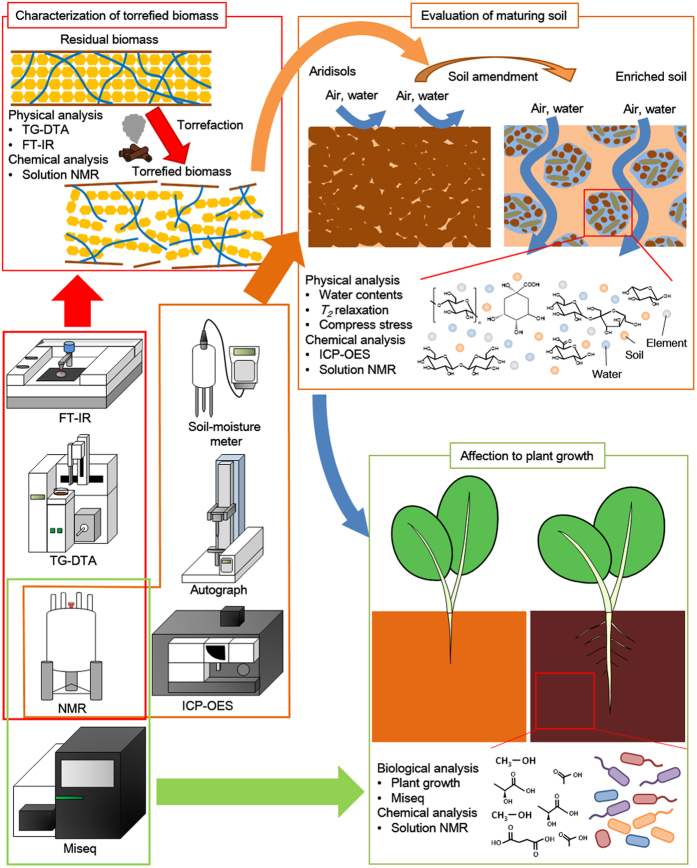
Schematic representation of this study. Effects of soil amendment with torrefied biomass were evaluated using three steps: (1) characterization of *Jatropha curcas* pyrolysis profiles, (2) evaluation of soil physical and chemical properties, and (3) evaluation of plant growth ability using *J. curcas* seedlings.

**Figure 2:**
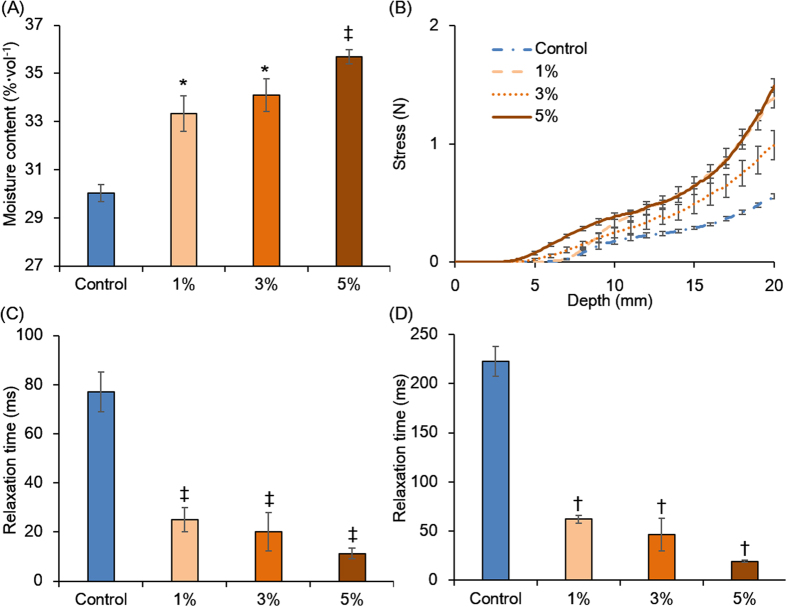
Physicochemical characterization of soils used for *Jatropha* cultivation with or without torrefied biomass. The moisture contents of saturated absorption (**A**) and compression mechanical stress under wet soil conditions (**B**). *T*_2_ relaxation time of binding (**C**) and free water contents (**D**). The error bars show the standard error of the mean and the *p* value for comparison of the control with each sample calculated using Welch’s *t* test. ^*^p < 0.05, ^†^*p* < 0.01, and ^‡^*p* < 0.005.

**Figure 3:**
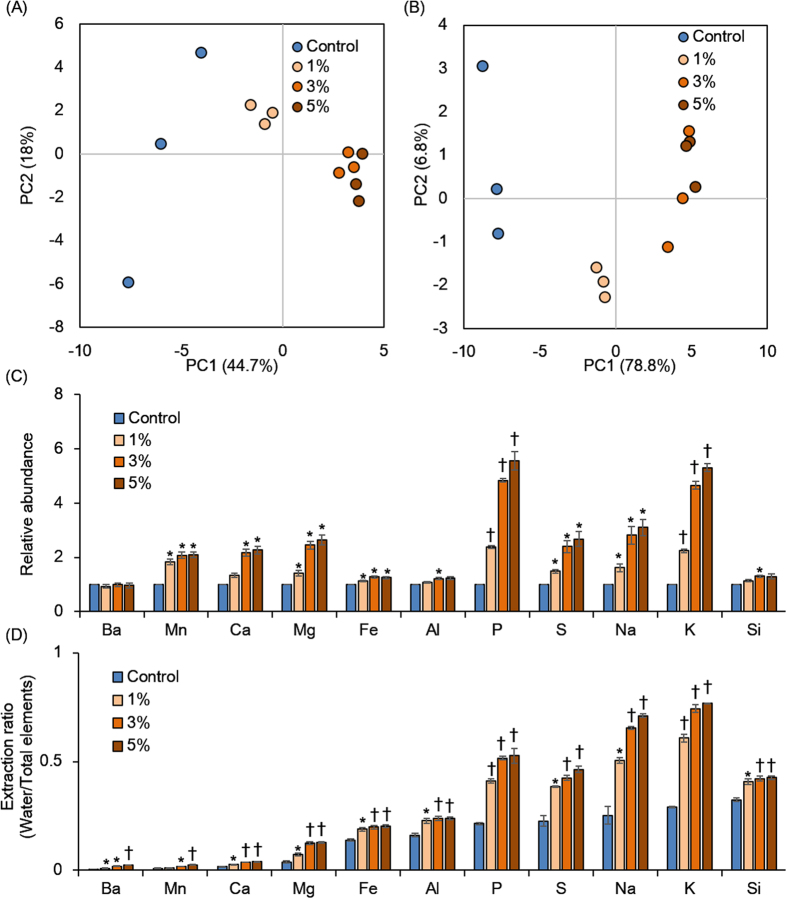
Evaluation of elemental components in soils. Soil elemental profiles were evaluated from extractions of water (**A**) and HNO_3_ (**B**) using PCA score plots and the relative abundance of soil total elements compared the control with torrefied biomass (**C**) and extraction ratios compared water with total extracted elements (**D**). The error bars show the standard error of the mean and the *p* value for comparison of the control with each sample calculated using Welch’s *t* test. ^∗^*p* < 0.05 and ^†^*p* < 0.01.

**Figure 4:**
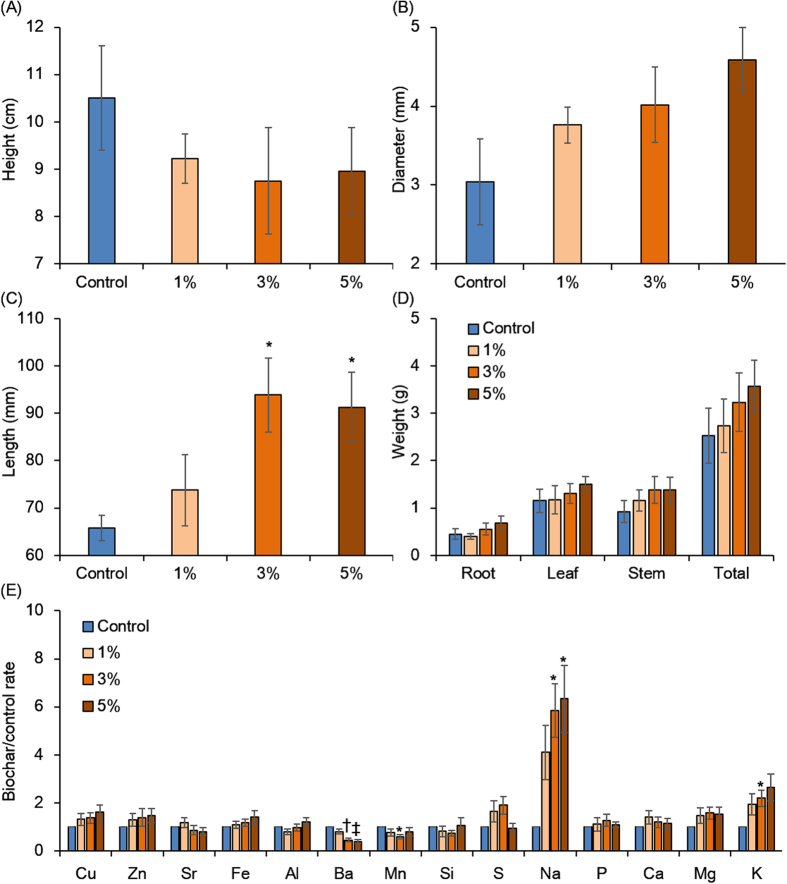
Evaluation of the effect of torrefied biomass on the initial plant growth stage. The effect of torrefied biomass on the plant phenotype during the initial growth stage was evaluated by plant height (**A**), stem diameter (**B**), root length (**C**), whole weight (**D**), and elemental content ratios of torrefied biomass to the control (**E**). The error bars show the standard error of the mean and the *p* value for comparison of the control with each sample, calculated using Welch’s *t* test. ^*^p < 0.05 and ^‡^*p* < 0.005.

**Figure 5:**
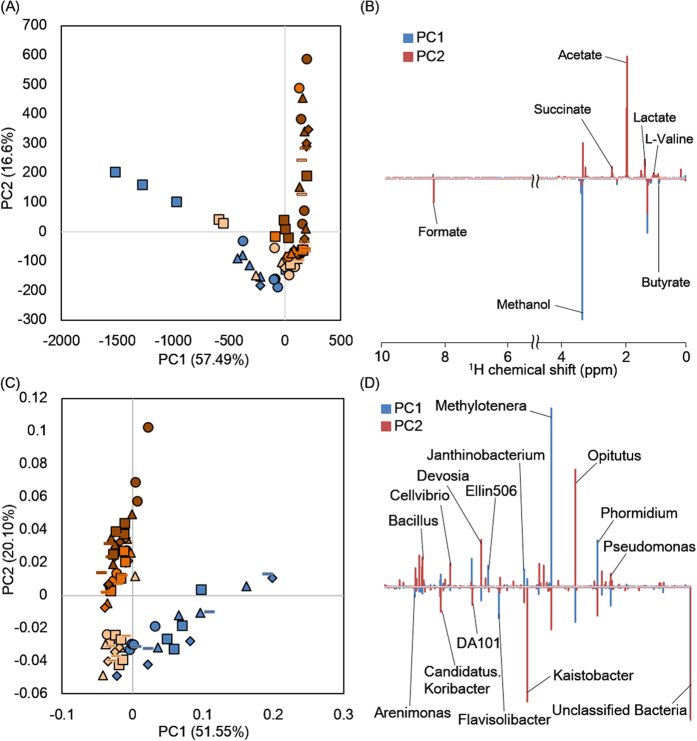
Soil metabolic and microbial profiles during plant growth. Metabolic (**A**,**B**) and microbial profiles (**C**,**D**) of soil during initial plant growth were evaluated using PCA score plots (**A**,**C**) and loading plots (**B**,**D**). Symbols in the score plot represent the control (blue), 1% (light orange), 3% (orange), and 5% torrefied biomass (brown) and weeks 0 (circle), 1 (triangle), 2 (diamond), 3 (square), and 4 (bar).

**Figure 6:**
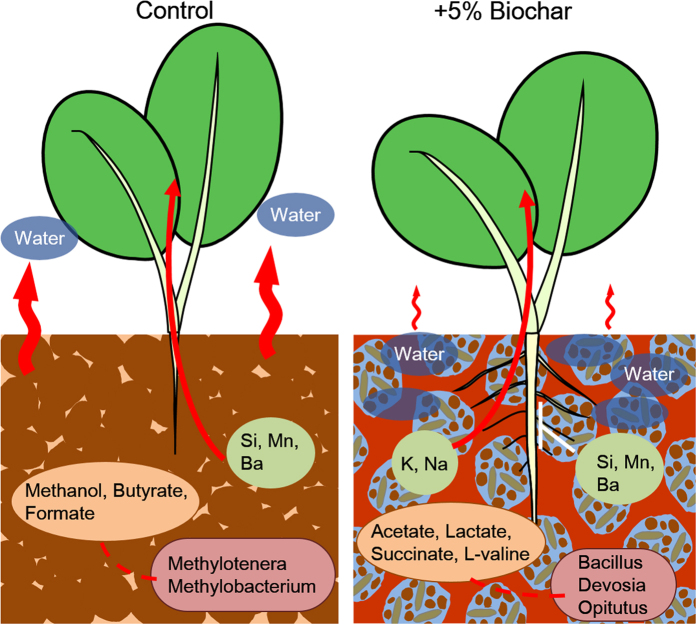
Schematic representation of the effects of incorporating torrefied biomass in soil on initial plant growth. Although the metabolic and microbial dynamics of the control were associated with a C1 metabolism (left), those of the 3–5% torrefied biomass samples were associated with an organic acid metabolism (right). This is attributed to the fact that torrefied biomass can improve physical and structural soil properties such as water retentivity and structural stability (right). Therefore, a soil amended with 3–5% of torrefied biomass can enhance the initial growth of *Jatropha curcas* in the form of increased stem diameter, root length, and element uptake ability.

The overall experimental design to evaluate the effects of soil amendments based on physical, chemical, and biological characteristics is illustrated in Fig. 1. For the torrefaction analysis, stem and leaf mixtures of *J. curcas* were milled with a food cutter, divided into 50-g samples, and wrapped in aluminum foil. The samples were torrefied at 200 °C, 220 °C, 230 °C, 240 °C, 250 °C, and 300 °C for 10 min under 5-L/min N_2_ in an electric furnace (FO410; Yamato Scientific Co., Ltd., Tokyo, Japan).

For growth experiments using *J. curcas*, a soil sample was collected from a *Jatropha* agricultural field in Gaborone, Botswana, in 2014. The soil was separated into four parts each weighing 3 kg into which 0, 30, 90, and 150 g of biomass torrefied at 240 °C [control, 1%, 3%, and 5% (weight/weight), respectively], and 150 g of fishmeal [5% (weight/weight)] was incorporated. To stabilize the metabolic activities and microbial variations in soils, they were incubated in a chamber at 25 °C and 10% moisture for 1 month and sampled twice a week. Seeds of *J. curcas* IP2P accession^39^ were germinated in 0.8 wt% agar gel with no nutrients as described in a previous study^40^. The germinated seeds after 10 days of growth were transplanted into the matured soils using quadruplicate experiments for the control and 1%, 3%, and 5% of torrefied biomass. *Jatropha* growth experiments with and without torrefied biomass were conducted over 4 weeks in a chamber at 25 °C and 50% moisture. Samples of 50 g were taken from the soils once a week for 4 weeks during growth.

### Characterization of torrefied biomass

A TG analysis was performed with an EXSTAR TG/DTA 6300 (SII Nanotechnology Inc., Tokyo, Japan) instrument following a previous study^23^. ATR FTIR was performed on a Nicolet 6700 FTIR (Thermo Fisher Scientific Inc., Waltham, MA, USA) instrument with a KBr disk following previous studies^23^,^41^. Grain size distribution was performed with a vibratory sieve shaker (Fritsch Japan Co., Ltd., Kanagawa, Japan) instrument, and the percentage was calculated for each weight.

### Soil characterization

For the analysis of soil compression stress, 9 g of dried soil samples was analyzed with an EZ-LX autograph (Shimazu, Kyoto, Japan) and TRAPEZIUM 2 software (Shimazu) at 5 mm/min to a depth of 20 mm and 25 N of stress using a lunge test jig. Soil samples with an addition of 2.5 ml ultrapure water were also measured under wet conditions using the same method. To measure the water content in soils, soil with or without 1%, 3%, or 5% torrefied biomass and raw biomass were divided into 600 g fractions and placed in plant pots into which 200 ml of ultrapure water was added. Soil water contents were measured with an ML3-theta probe soil moisture sensor (Delta-T Devices Ltd., Cambridge, England).

### Elemental analysis

For the ICP-OES analysis, 50 mg samples of Botswana soils with or without torrefied biomass and *J. curcas* in the initial growth stage were extracted with ultrapure water and then with HNO_3_ (6.9% v/v) following previous studies^42^,^30^ and analyzed using an ICP-OES instrument (SPS5510; SII Nanotechnology Inc., Tokyo, Japan).

### One- and two-dimensional NMR analyses

Samples of maturing phase soils with *Jatropha* growth (40 g) with an addition of sterile water were homogenized with a sonicator for 30 min and heated at 55 °C for 5 min in a Thermomixer comfort (Eppendorf AG, Hamburg, Germany). After centrifugation, the supernatants were collected and dried in a centrifugal evaporator (CVE-3000, Tokyo Rikakikai Co. Ltd., Tokyo, Japan). The dried samples were dissolved in 1 ml of D_2_O/KPi (100 mM, pH 7.0) and transferred into a 5 mm NMR tube.

The torrefied biomass and soil after *Jatropha* growth were analyzed using ^1^H-^13^C HSQC to identify the components. NMR spectra were acquired at 25 °C using an AVANCE II 700 MHz Bruker Biospin (Rheinstetten, Germany) instrument equipped with an inverse (with proton coils nearest to the sample) 5 mm ^1^H/^13^C/^15^N cryoprobe. The peak of sodium 2,2-dimethyl-2-silapentane-5-sulfonate (DSS) was used as the internal reference (calibrated at δ_C_ 140, δ_H_ 0 ppm). NMR spectra were acquired from 11.704 to −2.296 ppm in F2 (^1^H) using 2048 data points for an acquisition time of 104 ms, recycling delay of 2 s, and 150−10 ppm in F1 (^13^C) using 256 data points of 48 scans. All one-dimensional Watergate and two-dimensional (2D) J-resolved (2D-J) spectra were acquired with the same NMR instrument to evaluate metabolic profiles of soil microbiota. Watergate spectra were measured from 14 to −3 ppm at 25 °C using 32 k data points. 2D-J spectra were acquired from 11.7568 to −2.2458 ppm in F2 (^1^H) using 16 k data points and from 20.0027 to −19.9973 Hz in F1 (*J* coupling) using 16 data points of eight scans. HSQC and 2D-J spectra were further analyzed for annotation of chemical components using SpinAssign (http://prime.psc.riken.jp)^43^,^44^, Biological Magnetic Resonance Bank (http://www.bmrb.wisc.edu/)^45^, and Birmingham Metabolite Library (http://www.bml-nmr.org/)^46^.

Relaxation time analyses of the water content in soils were measured by solid-state NMR using a Bruker DRX-500 spectrometer operating at 500.13 MHz for ^1^H equipped with the Bruker 4 mm double-tuned MAS probe. For the NMR measurements, approximately 80 mg of a sample and 100 μl of sterilized water were placed in a ZrO_2_ rotor (outer diameter 4 mm) with a Kel-F cap. The magic angle (54.7°) pulse length for protons was set to 1.8 μs. The measurement program used 2D Carr Purcell Meiboom Gill and sampling of the decay/recovery curves was obtained at 2–80 ms.

### Metasequencing

Microbial DNA extraction was performed using the PowerSoil^™^ DNA Isolation Kit (Mo Bio Laboratories Inc., Carlsbad, CA, USA) according to the manufacturer’s instructions. The polymerase chain reaction (PCR) protocol used for metasequencing was described previously^47^,^48^. Sequencing was performed on a MiSeq sequencer (Illumina, San Diego, CA, USA) according to the manufacturer’s instructions. The data were analyzed using QIIME software (http://qiime.org/)^49^. Each sample was separated by a MiSeq barcode-attached 27F mod-534R primer and chimera check using Usearch software (http://drive5.com/usearch/manual/uchime_algo.html)^50^. The resulting operational taxonomic unit data defined over 97% similarity were assigned to sequences using the Ribosomal Database Project (http://rdp.cme.msu.edu/seqmatch/seqmatch_intro.jsp) classifier^51^.

### Statistical analysis

All ^1^H-NMR data were processed using Topspin 3.1 software (Bruker Biospin), and raw data were exported as text files. Exported data were processed over a range of 11 to −1 ppm with approximately 27.5 k data points for ^1^H-NMR and binning using R 3.0.1 software (http://www.r-project.org/). The dataset was normalized using the sum of the DSS integral regions and analyzed by PCA using R software as previously described^26^,^33^,^52^.

## Additional Information

**How to cite this article**: Ogura, T. *et al.* Improvement of physical, chemical, and biological properties of aridisol from Botswana by the incorporation of torrefied biomass. *Sci. Rep.* **6**, 28011; doi: 10.1038/srep28011 (2016).

## Supplementary Material

Supplementary Information
