# Supplementary material for: Improvement of physical, chemical, and biological properties of aridisol from Botswana by the incorporation of torrefied biomass
Source: Sci Rep. 2016 Jun 17;6:28011. doi: 10.1038/srep28011 (PMC4911548; doi:10.1038/srep28011)
Supplement: Supplementary Information [file srep28011-s1.doc]

**Improvement of physical, chemical, and biological properties of aridisol from Botswana by the incorporation of torrefied biomass**

Tatsuki Ogura, ^†,‡^ Yasuhiro Date, ^†,‡^ Masego Masukujane,^§^ Tidimalo Coetzee,^§^ Kinya Akashi,^‖^ and Jun Kikuchi*^,†,‡,¶^

^†^RIKEN Center for Sustainable Resource Science, 1-7-22 Suehiro-cho, Tsurumi-ku, Yokohama 230-0045, Japan

^‡^Graduate School of Medical Life Science, Yokohama City University, 1-7-29 Suehiro-cho, Tsurumi-ku, Yokohama 230-0045, Japan

^§^Department of Agricultural Research, Ministry of Agriculture, Private Bag 0033, Gaborone, Botswana

^‖^Faculty of Agriculture, Tottori University, 4-101 Koyama-cho, Tottori 680-8533, Japan

^¶^Graduate School of Bioagricultural Sciences, Nagoya University, 1 Furo-cho, Chikusa-ku, Nagoya 464-0810, Japan

*Corresponding author:

Jun Kikuchi

RIKEN Center for Sustainable Resource Science

1-7-22 Suehiro-cho, Tsurumi-ku, Yokohama 230-0045, Japan

Phone: +81455039490;

Fax: +81455039489;

E-mail:jun.kikuchi@riken.jp

Number of pages: 12

Number of figures: 11

Number of tables: 3

### Supporting Information

**Contents**

**Table S1.** Annotation list of Fourier transform infrared spectra. **(Page S3)**

**Table S2.** Annotation list of ^1^H-^13^C heteronuclear single quantum coherence spectra. **(Page S3)**

**Table S3.** Annotation list of two-dimensional J-resolved nuclear magnetic resonance spectra. **(Page S6)**

**Figure S1.** Pyrodegradation profiles of *Jatropha curcas* derived from the thermogravimetric differential thermal analysis measurements. **(Page S7)**

**Figure S2.** Compositional characterization of torrefied biomass treated at different temperatures measured by Fourier transform infraredspectra. **(Page S7)**

**Figure S3.** Grain size distribution of torrefied biomass measured by vibratory sieve shaker. **(Page S8)**

**Figure S4.** Characterization of KPi/D_2_O soluble components degraded by torrefaction by ^1^H-^13^C heteronuclear single quantum coherence spectra. **(Page S8)**

**Figure S5.** *T*_2_ relaxation curve of water in soils with and without torrefied biomass. **(Page S9)**

**Figure S6.** Water retentivity of soils used for *Jatropha* cultivation with or without raw biomass. The error bars show the standard error of the mean and the *p* value for comparison of the control with each sample, calculated using Welch’s *t* test. ^∗^*p* < 0.05 and ^‡^*p* < 0.005. **(Page S9)**

**Figure S7.** Metabolic profiles of maturing soils analyzed by time series of ^1^H-nuclear magnetic resonance spectra. **(Page S10)**

**Figure S8.** Metabolic profiles of soils with torrefied biomass or fishmeal during the maturing phase evaluated using a PCA score plot (**A**) and a loading plot (**B**). **(Page S10)**

**Figure S9.** Two-dimensional J-resolved nuclear magnetic resonance spectrum of 5% torrefied biomass soil after 1 week of plant growth. **(Page S11)**

**Figure S10.** Metabolite dynamics during plant growth phase (0–4 weeks) versus annotated peak intensities in ^1^H-nuclear magnetic resonance spectra. **(Page S11)**

**Figure S11.** Microbial dynamics during plant growth (0–4 weeks) versus the microbial abundance rate among the total MiSeq sequencing data. **(Page S12)**

**Table S1.** Annotation list of Fourier transform infrared spectra

| No. | Wave number (cm^−1^) | Annotation |
| --- | --- | --- |
| 1 | 895 | Anomeric vibration at β-glycoside linkage |
| 2 | 1,032 | C–O stretching in cellulose and hemicellulose |
| 3 | 1,153 | Deformation vibration of C–H bonds in benzene rings |
| 4 | 1,230 | Syringyl ring and C–O stretching in lignin and xylan |
| 5 | 1,317 | C–H in cellulose and C_1_–O vibration in syringyl derivative |
| 6 | 1,373 | C–H deformation in cellulose and hemicellulose |
| 7 | 1,450 | Asymmetric C–H bonding in CH_3_ and -CH_2_- |
| 8 | 1,516 | Aromatic ring vibrations |
| 9 | 1,608 | Aromatic ring vibrations and C=O stretching |
| 10 | 1,728 | Stretching of C=O unconjugated to aromatic rings (oxidized side chains) |
| 11 | 2,927 | C–H stretching |
| 12 | 3,149–3,453 | O–H stretching |

**Table S2.** Annotation list of ^1^H-^13^C heteronuclear single quantum coherence spectra

|  | δ_H_ (ppm) | δ_C_ (ppm) | Annotation | | | |
| --- | --- | --- | --- | --- | --- | --- |
| 1 | 1.304 | 22.9 | Lactate |  |  |  |
| 2 | 1.523 | 28.655 | Adipate |  |  |  |
| 3 | 1.864 | 43.522 | Quinate |  |  |  |
| 4 | 1.892 | 26.3 | Acetate |  |  |  |
| 5 | 1.953 | 40.206 | Quinate |  |  |  |
| 6 | 2.036 | 40.214 | Quinate |  |  |  |
| 7 | 2.05 | 43.521 | Quinate |  |  |  |
| 8 | 2.122 | 23.3 | *O*-acetylcarnitine |  |  |  |
| 9 | 2.124 | 29.052 | l-Glutamate | l-Glutamine |  |  |
| 10 | 2.155 | 40.439 | Adipate |  |  |  |
| 11 | 2.392 | 37.009 | Succinate |  |  |  |
| 12 | 2.394 | 45.39 | Malate |  |  |  |
| 13 | 2.438 | 33.644 | l-Glutamine |  |  |  |
| 14 | 2.68 | 45.45 | Malate |  |  |  |
| 15 | 3.184 | 56.685 | Choline |  |  |  |
| 16 | 3.224 | 43.299 | l-Arginine | Tyramine |  |  |
| 17 | 3.23 | 76.956 | d-Glucose | Gentiobiose |  |  |
| 18 | 3.266 | 77.161 | Maltose |  |  |  |
| 19 | 3.392 | 72.422 | d-Glucose | Gentiobiose | Maltose |  |
| 20 | 3.457 | 72.013 | Gentiobiose | Maltodextrin | Sucrose |  |
| 21 | 3.46 | 78.657 | d-Glucose | Gentiobiose |  |  |
| 22 | 3.5 | 70.227 | Choline |  |  |  |
| 23 | 3.536 | 78.037 | Quinate |  |  |  |
| 24 | 3.541 | 73.88 | d-Glucose | Gentiobiose | Sucrose |  |
| 25 | 3.543 | 65.408 | d-Fructose | Glycerol |  |  |
| 26 | 3.545 | 66.661 | d-Fructose |  |  |  |
| 27 | 3.618 | 74.33 | Maltodextrin |  |  |  |
| 28 | 3.635 | 72.547 | d-Galactono-1,4-lactone |  |  |  |
| 29 | 3.64 | 65.219 | d-Fructose | Glycerol | Threonate |  |
| 30 | 3.647 | 79.613 | Maltodextrin | Maltose |  |  |
| 31 | 3.664 | 64.135 | Sucrose |  |  |  |
| 32 | 3.689 | 77.967 | d-Galactose |  |  |  |
| 33 | 3.697 | 66.702 | d-Fructose |  |  |  |
| 34 | 3.697 | 75.593 | d-Glucose | d-Glucuronate | Gentiobiose | Maltodextrin |
|  |  |  | Maltose |  |  |  |
| 35 | 3.704 | 63.56 | d-Glucose | Gentiobiose |  |  |
| 36 | 3.733 | 73.257 | d-Arabitol | Mannitol |  |  |
| 37 | 3.748 | 75.362 | Sucrose |  |  |  |
| 38 | 3.761 | 57.005 | l-Arginine | l-Glutamate | l-Glutamine |  |
| 39 | 3.783 | 70.35 | d-Fructose |  |  |  |
| 40 | 3.802 | 62.923 | Sucrose |  |  |  |
| 41 | 3.804 | 65.175 | d-Fructose | Sucrose |  |  |
| 42 | 3.814 | 83.475 | d-Fructose |  |  |  |
| 43 | 3.818 | 74.238 | d-Glucose |  |  |  |
| 44 | 3.829 | 75.22 | Sucrose |  |  |  |
| 45 | 3.877 | 84.162 | Sucrose |  |  |  |
| 46 | 3.88 | 72.489 | d-Fructose |  |  |  |
| 47 | 3.881 | 63.548 | d-Glucose | Gentiobiose | Maltose |  |
| 48 | 3.97 | 75.618 | Threonate |  |  |  |
| 49 | 4.003 | 69.839 | Quinate |  |  |  |
| 50 | 4.011 | 66.185 | d-Fructose |  |  |  |
| 51 | 4.037 | 76.784 | Sucrose |  |  |  |
| 52 | 4.042 | 84.12 | d-Fructose |  |  |  |
| 53 | 4.044 | 58.42 | Choline |  |  |  |
| 54 | 4.046 | 74.994 | d-Glucuronate |  |  |  |
| 55 | 4.069 | 73.294 | d-Galactose |  |  |  |
| 56 | 4.096 | 84.767 | d-Fructose |  |  |  |
| 57 | 4.097 | 78.192 | d-Fructose |  |  |  |
| 58 | 4.098 | 77.237 | d-Fructose |  |  |  |
| 59 | 4.127 | 73.239 | Quinate |  |  |  |
| 60 | 4.201 | 79.199 | Sucrose |  |  |  |
| 61 | 4.309 | 73.096 | Malate |  |  |  |
| 62 | 4.626 | 98.68 | d-Glucose | d-Glucuronate | Gentiobiose |  |
| 63 | 5.217 | 94.909 | α-d-Galactose | d-Glucose | d-Glucuronate | Gentiobiose |
|  |  |  | Maltose |  |  |  |
| 64 | 5.391 | 102.447 | Maltodextrin | Maltose |  |  |
| 65 | 5.398 | 94.995 | Sucrose |  |  |  |
| 66 | 6.883 | 118.724 | Tyramine |  |  |  |

**Table S3.** Annotation list of two-dimensional J-resolved nuclear magnetic resonance spectra

| δ_H_ (ppm) | J couple (Hz) | | Coupling pattern | Annotation |
| --- | --- | --- | --- | --- |
| 8.45 |  |  | Singlet | Formate |
| 4.11 | 3.53 |  | Doublet | Lactate |
| 3.35 |  |  | Singlet | Methanol |
| 2.15 | 7.01 |  | Triplet | Butyrate |
| 1.91 |  |  | Singlet | Acetate |
| 1.57 | 10.97 | 3.39 | Quartet | Butyrate |
| 1.32 | 3.44 | 1.09 | Quartet | Lactate |
| 1.05 | 3.57 |  | Doublet | Valine |
| 0.99 | 3.33 |  | Doublet | Valine |
| 0.89 | 7.35 |  | Triplet | Butyrate |

**
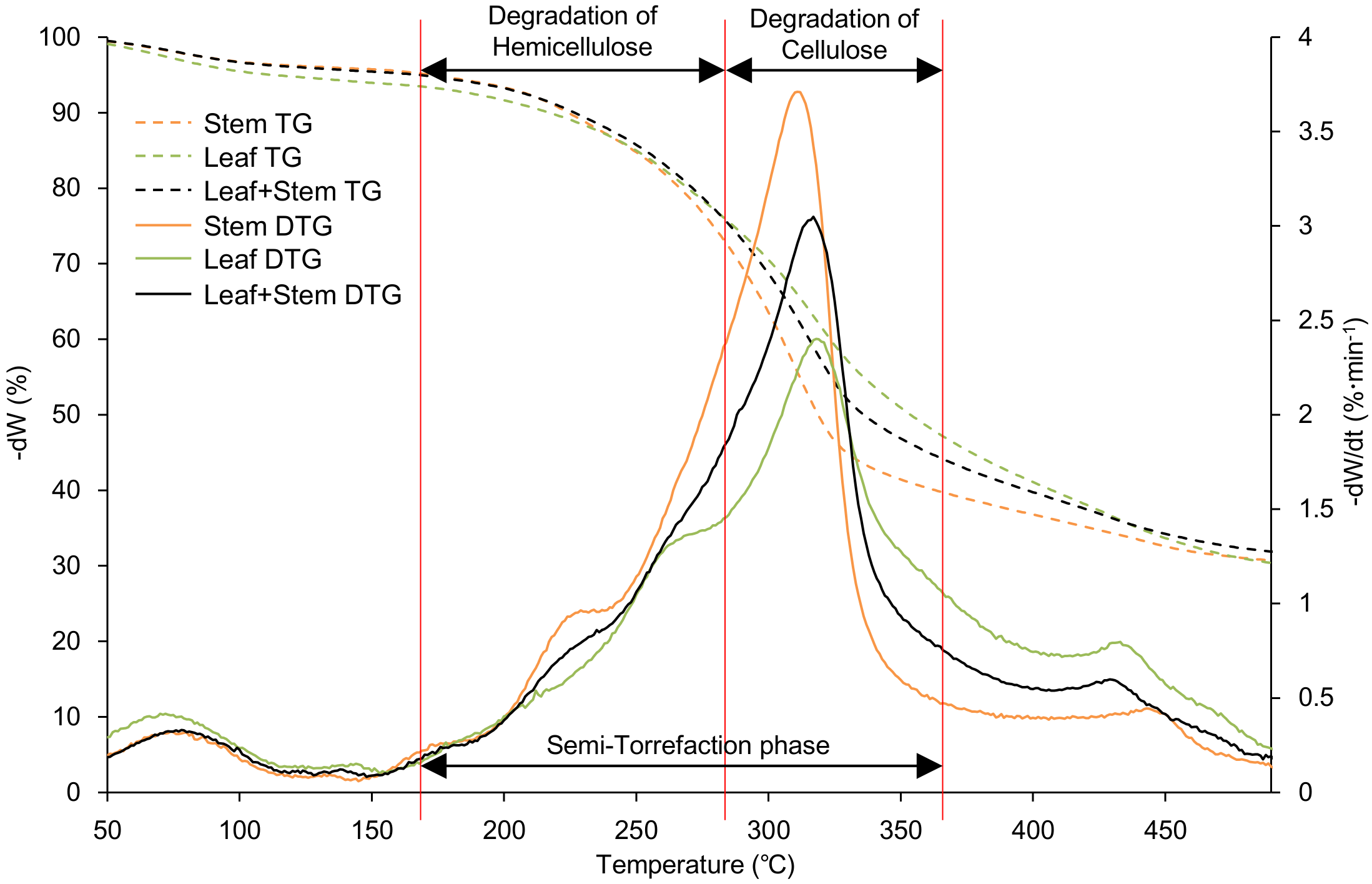
**

**Figure S1.** Pyrodegradation profiles of *Jatropha curcas* derived from the thermogravimetric differential thermal analysis (TG-DTA) measurements. Green, orange, and black lines represent degradation profiles of stems, leaves, and their mixture, respectively. The broken lines represent TG, and the solid lines represent DTA.


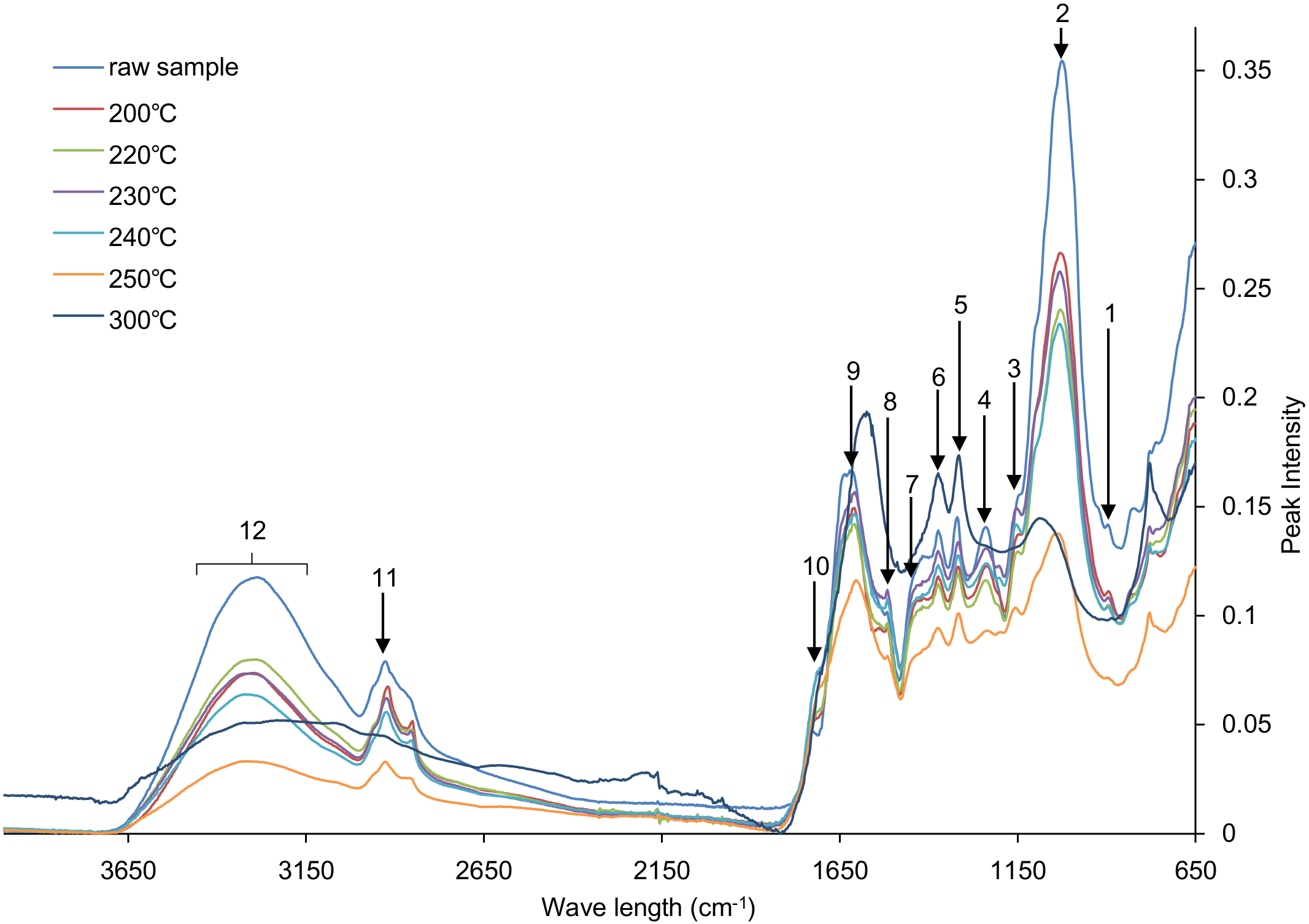


**Figure S2.** Compositional characterization of torrefied biomass treated at different temperatures measured by Fourier transform infrared spectra. Numerical annotations are described in Table S1.


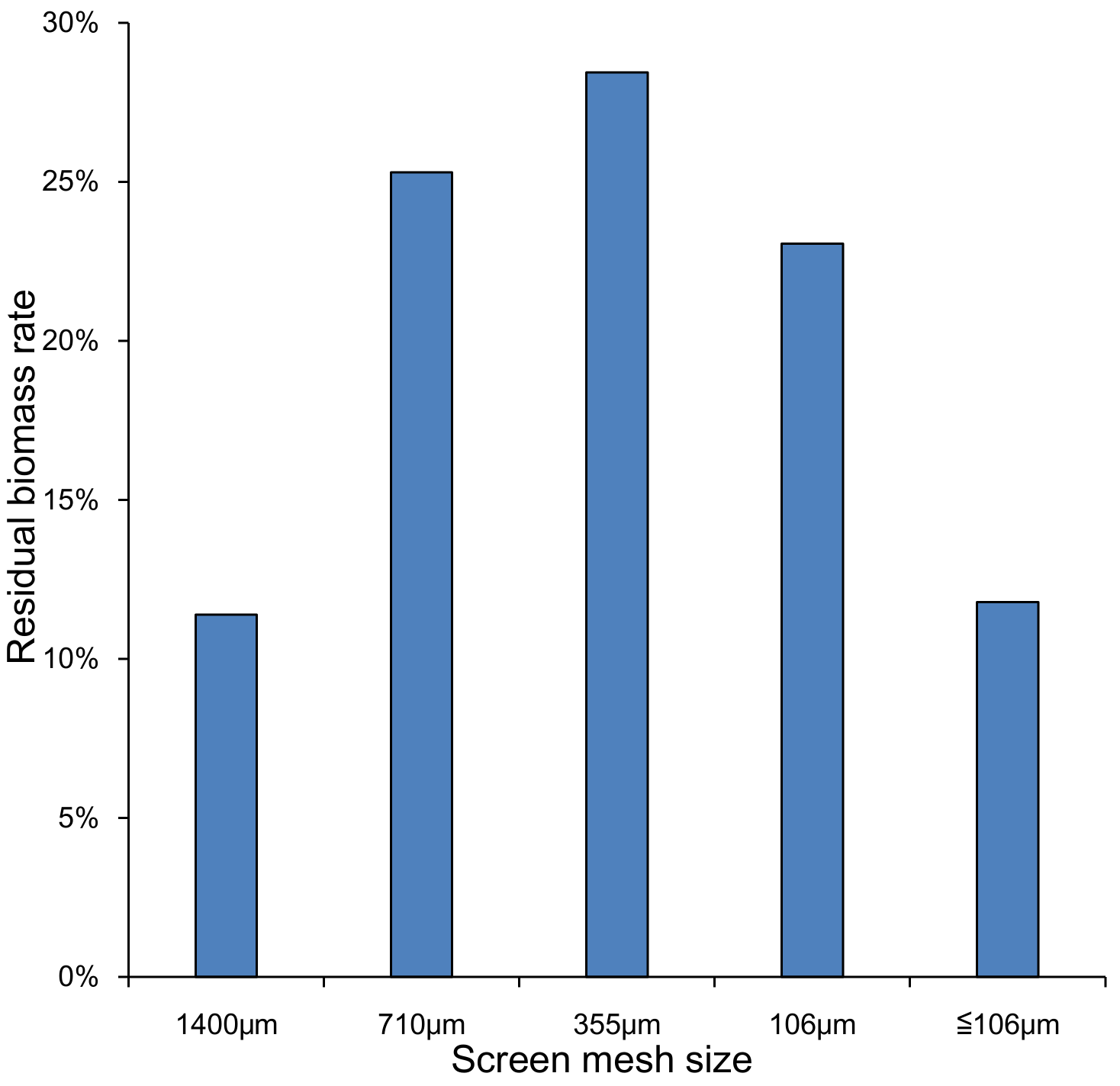


**Figure S3.** Grain size distribution of torrefied biomass measured by vibratory sieve shaker.


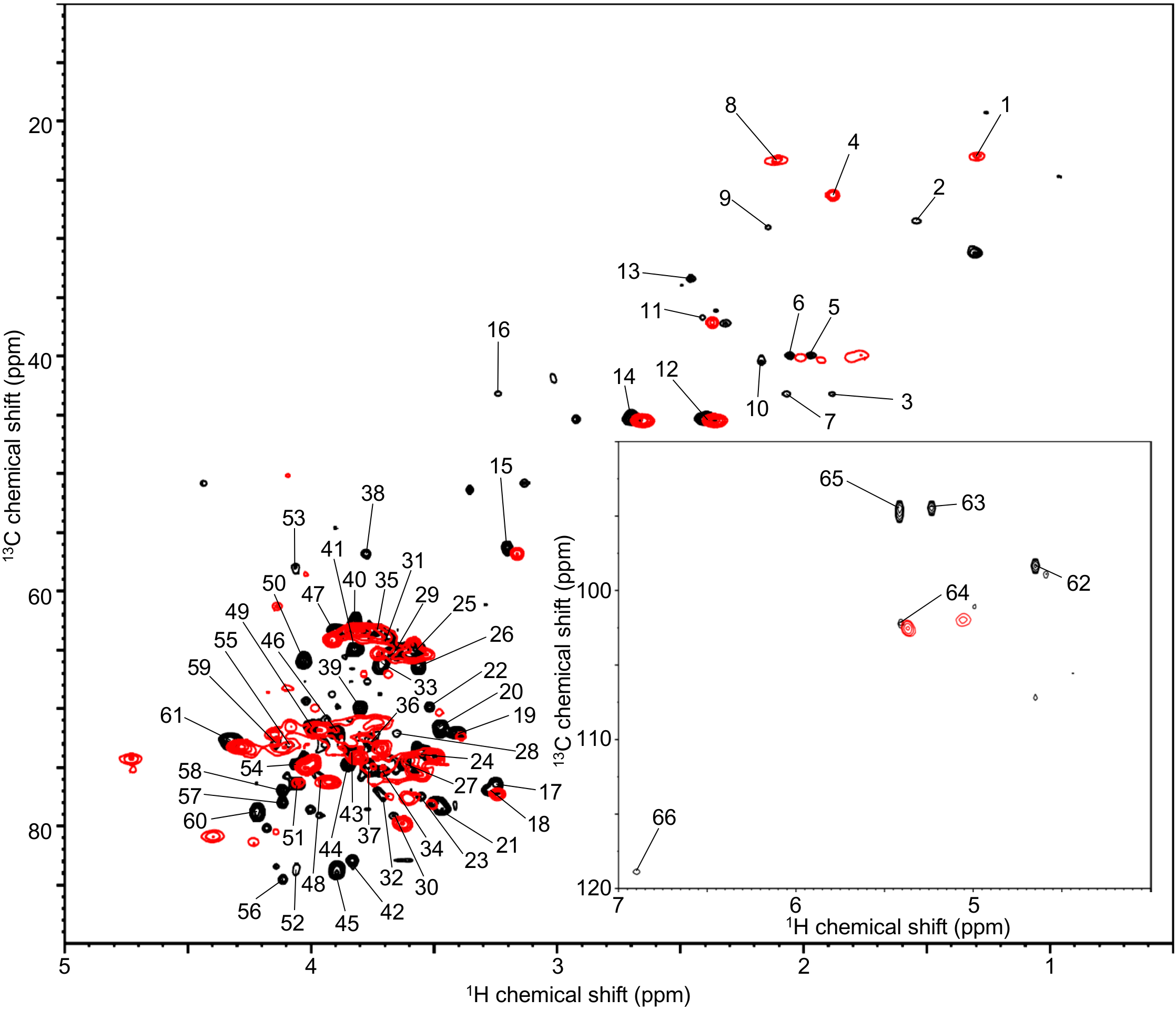


**Figure S4.** Characterization of KPi/D_2_O soluble components degraded by torrefaction by ^1^H-^13^C heteronuclear single quantum coherence spectra. The black and red spectra indicate raw samples and samples torrefied at 240°C, respectively. The numbers indicate annotated metabolites listed in Table S2.


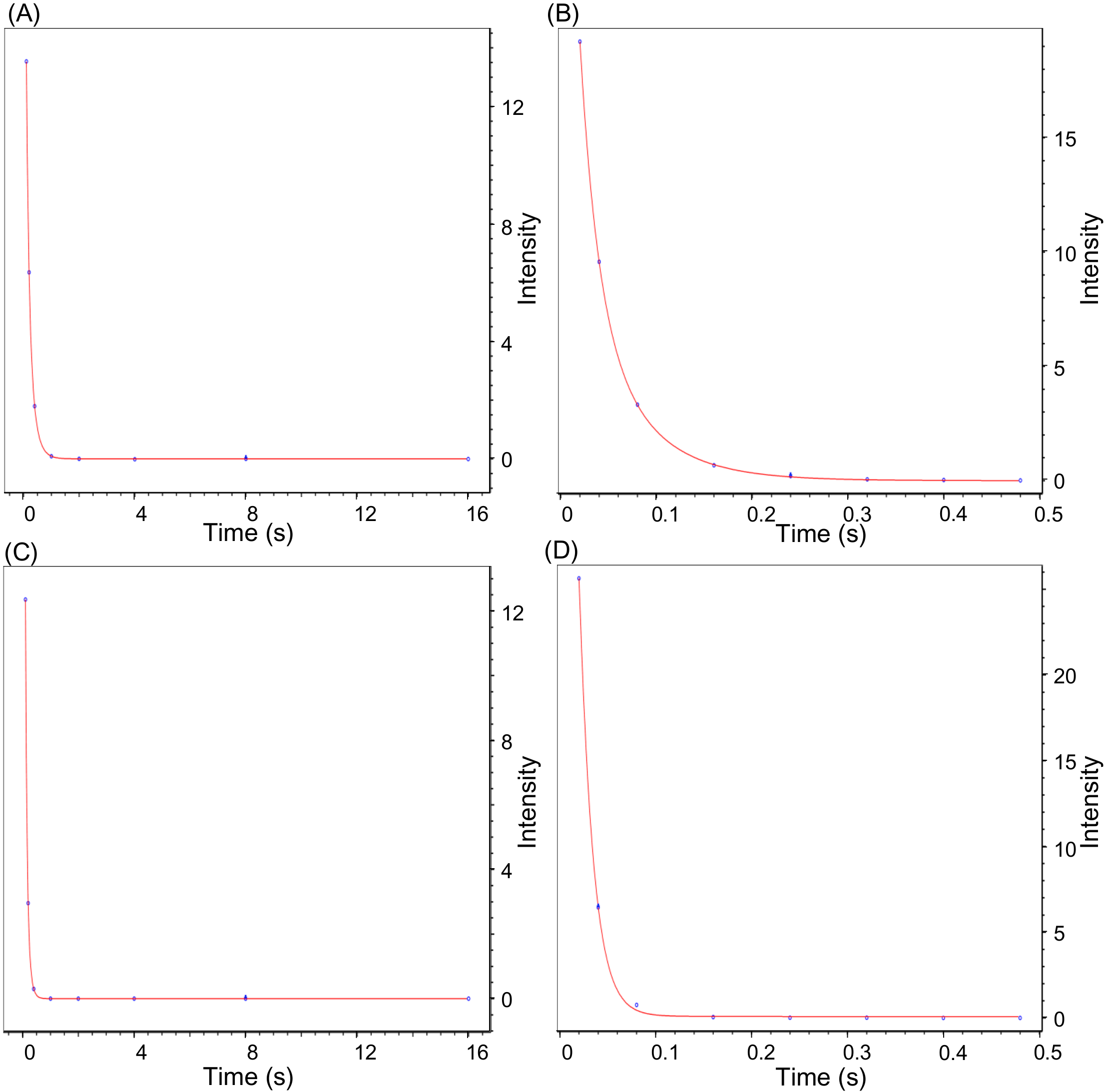


**Figure S5.** *T_2_* relaxation curve of water in soils with and without torrefied biomass. (**A**) Control, (**B**) 1%, (**C**) 3%, and (**D**) 5% torrefied biomass.


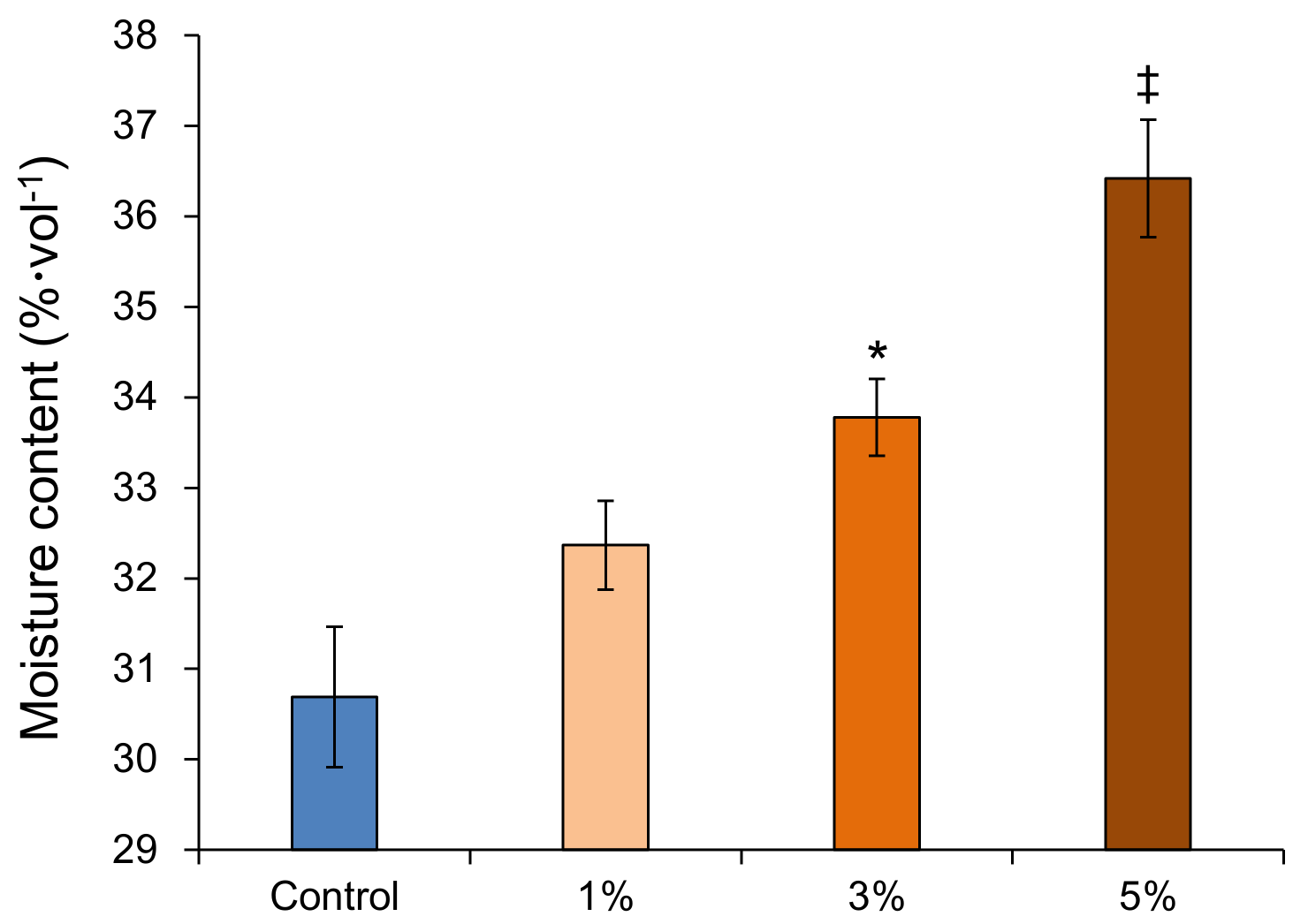


**Figure S6.** Water retentivity of soils used for *Jatropha* cultivation with or without raw biomass. The error bars show the standard error of the mean and the *p* value for comparison of the control with each sample calculated using Welch’s *t* test. ^∗^*p* < 0.05 and ^‡^*p* < 0.005.


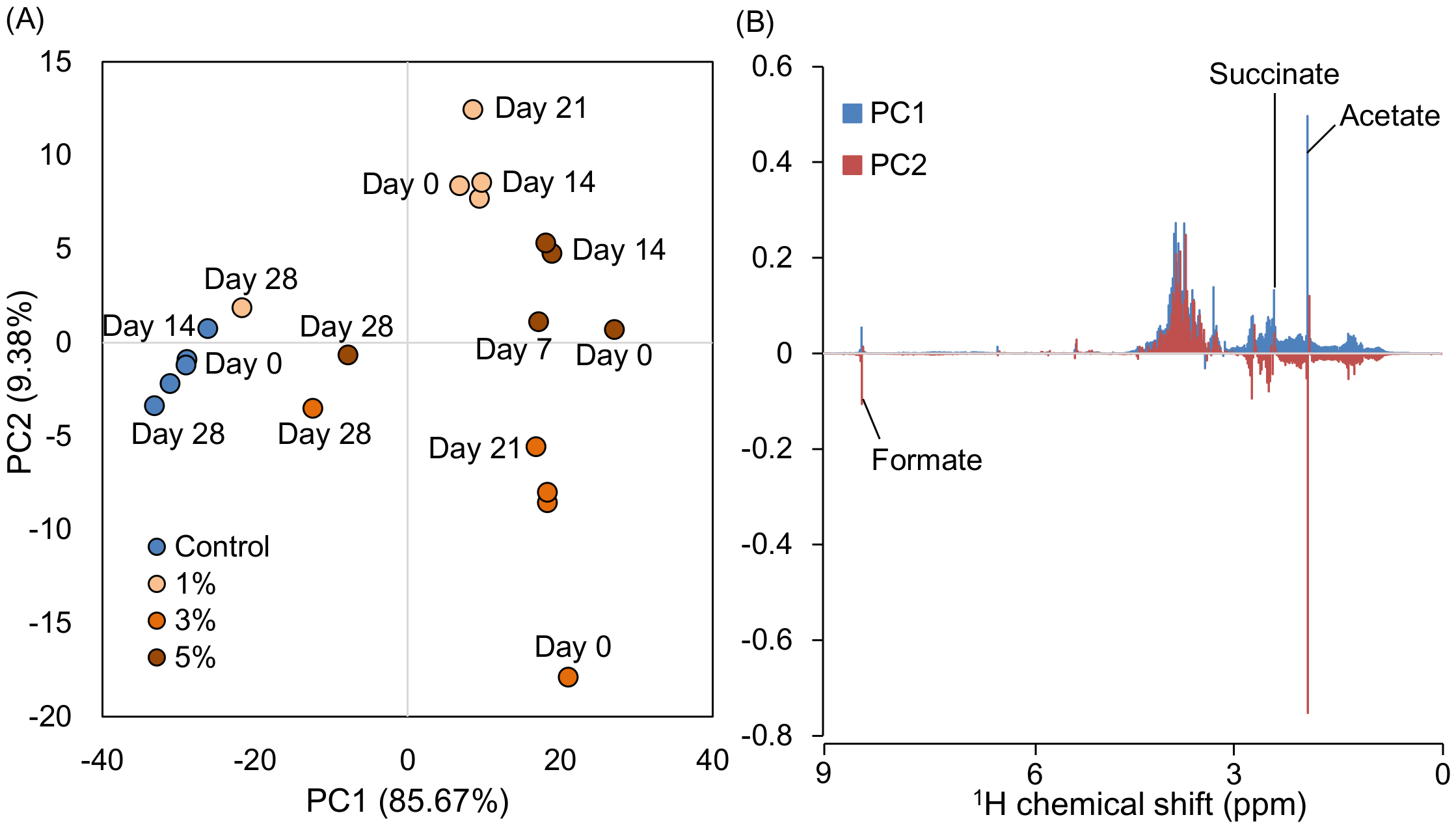


**Figure S7.** Metabolic profiles of maturing soils analyzed by a time series of ^1^H-nuclear magnetic resonance spectra. Metabolic dynamics of soils with or without torrefied biomass during the maturing phase were evaluated using a PCA score plot (**A**) and a loading plot (**B**).


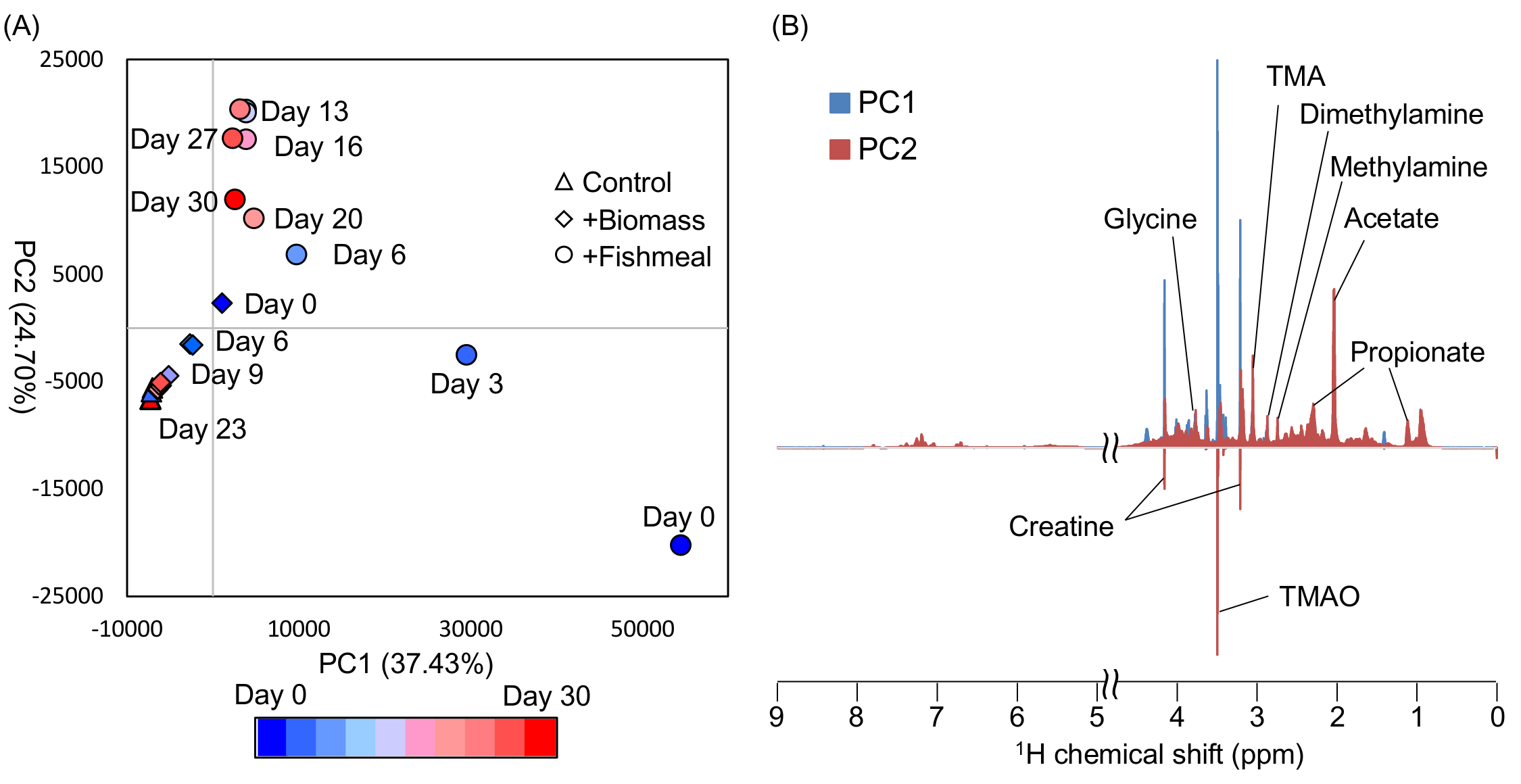


**Figure S8.** Metabolic profiles of soils with torrefied biomass or fishmeal during the maturing phase evaluated using a PCA score plot (**A**) and a loading plot (**B**).


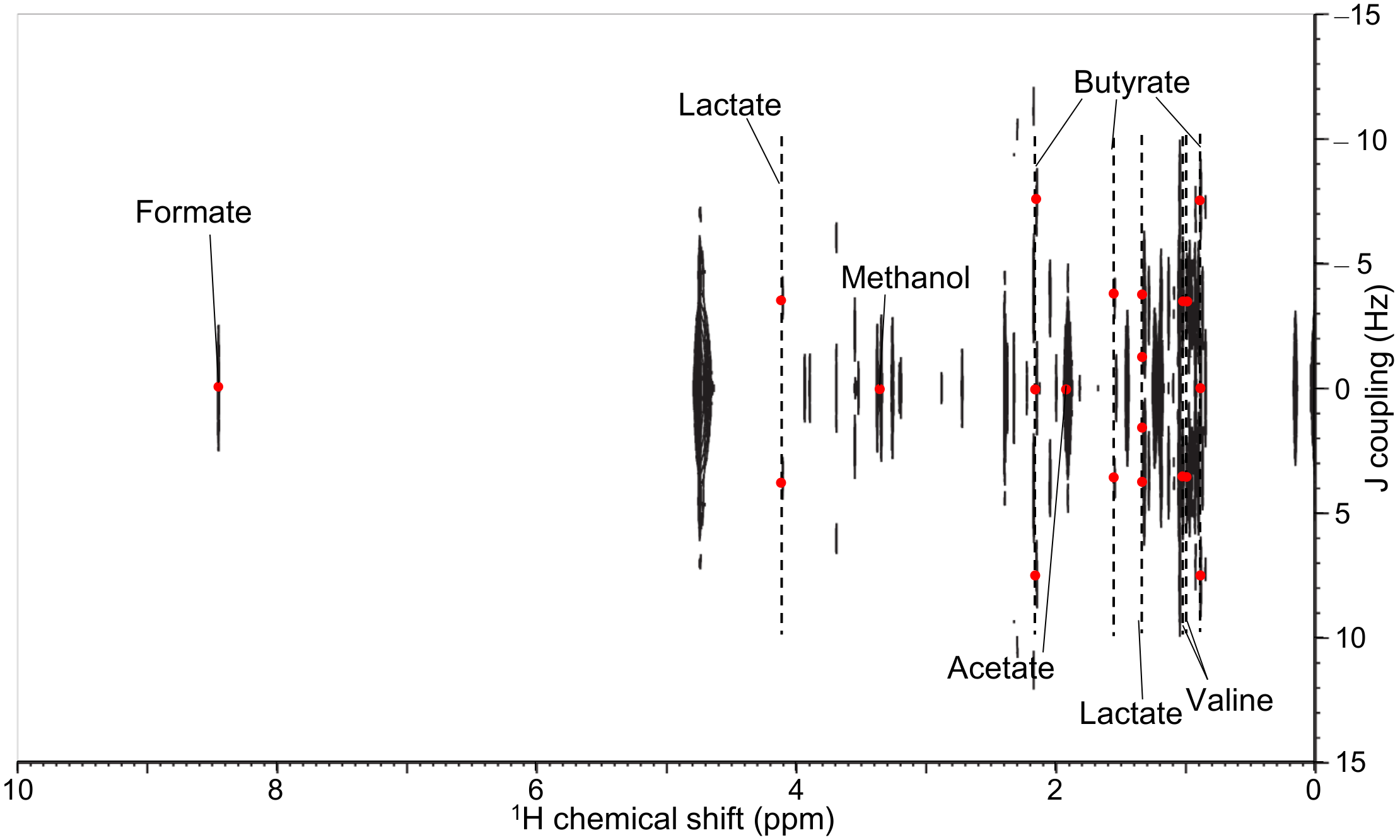


**Figure S9.** Two-dimensional J-resolved nuclear magnetic resonance spectrum of 5% torrefied biomass soil after 1 week of plant growth. The annotations of typical compounds in C1 and organic acid metabolisms in soils referred to the Birmingham Metabolite Library and are described in Table S3.


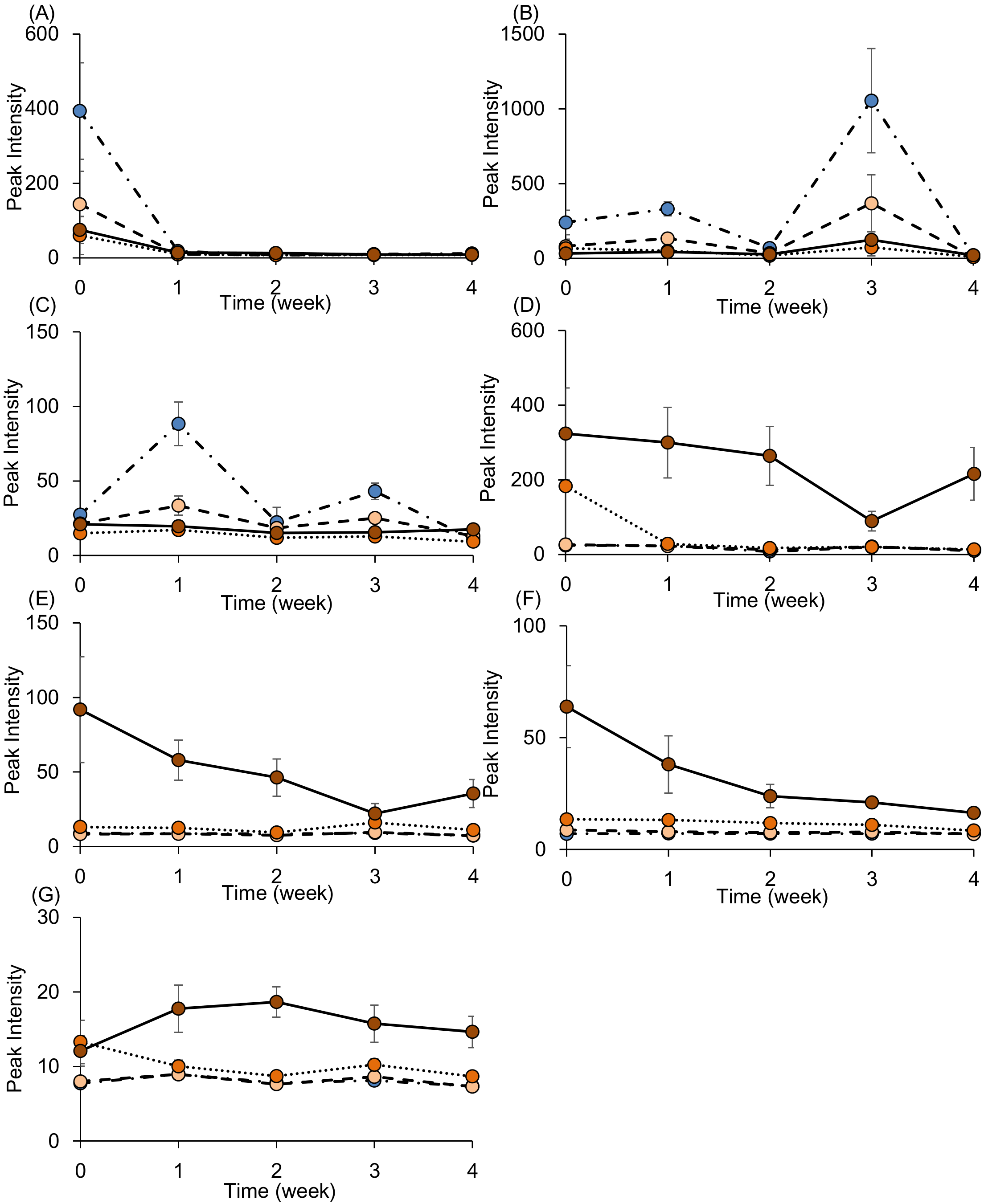


**Figure S10.** Metabolite dynamics during the plant growth phase (0–4 weeks) versus annotated peak intensities in ^1^H-nuclear magnetic resonance spectra. (**A**) Formate, (**B**) methanol, (**C**) butyrate, (**D**) acetate, (**E**) lactate, (**F**) succinate, and (**G**) l-valine. Symbols refer to the control (blue), 1% (light orange), 3% (orange), and 5% torrefied biomass (brown).


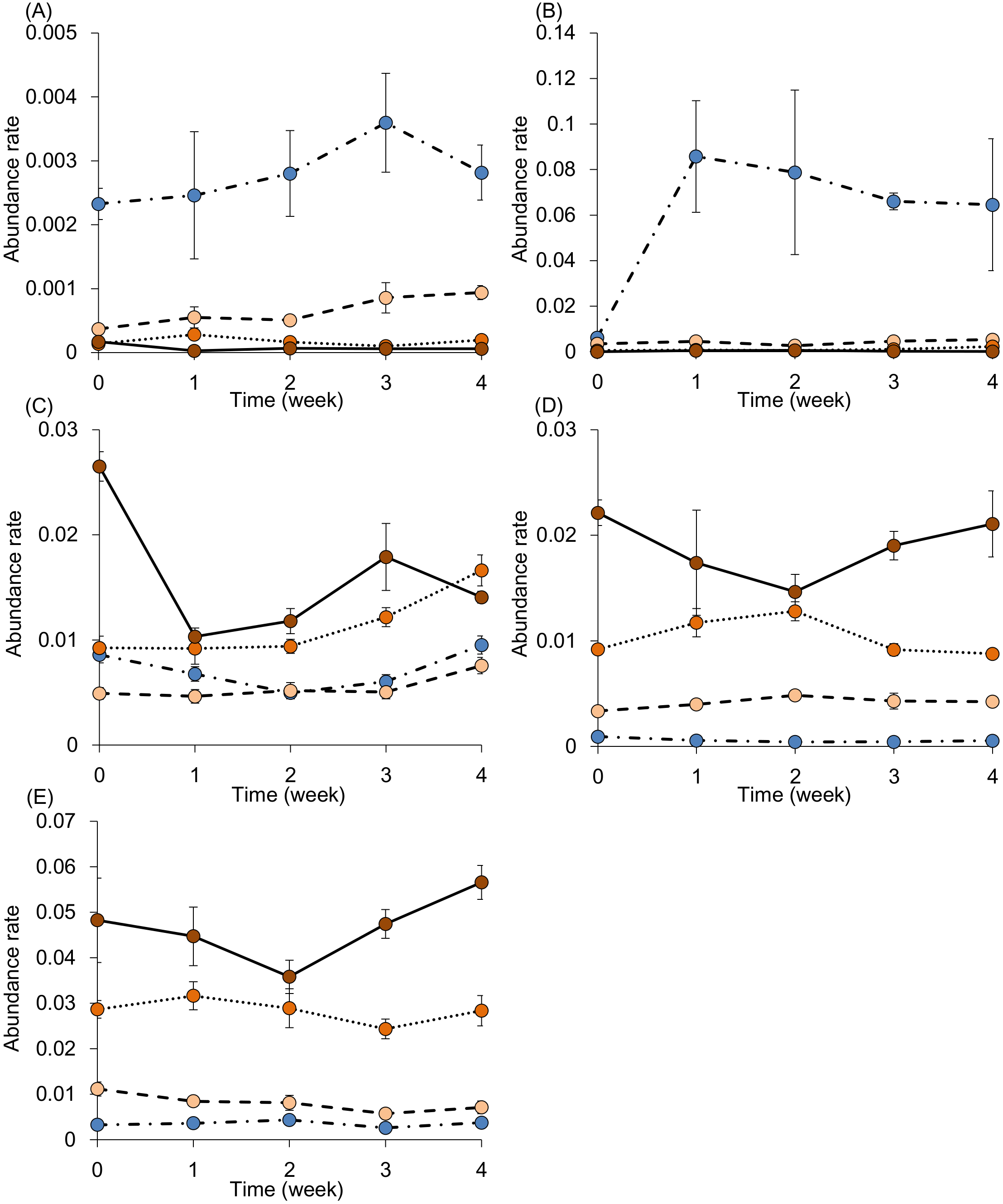


**Figure S11.** Microbial dynamics during plant growth (0–4 weeks) versus the microbial abundance rate among the total MiSeq sequencing data. (**A**) *Methylobacterium* sp., (**B**) *Methylotenera* sp., (**C**) *Bacillus* sp., (**D**) *Devosia* sp., and (**E**) *Opitutus* sp. Symbols refer to the control (blue), 1% (light orange), 3% (orange), and 5% torrefied biomass (brown).
